# Four-year review of presenteeism data among employees of a large United States health care system: a retrospective prevalence study

**DOI:** 10.1186/s12960-018-0321-9

**Published:** 2018-11-09

**Authors:** Donna Allen, Erica Wandtke Hines, Vanessa Pazdernik, Lynda Tierney Konecny, Erin Breitenbach

**Affiliations:** 1 College of Graduate Health Studies, A.T. Still University, Kirksville, MO USA; 2 Research Support, A.T. Still University, Kirksville, MO USA; 3 Department of Public Health, School of Nursing and Health SciencesCapella University, Kirksville, USA

**Keywords:** Presenteeism, Employees, Health care system, Human resources, Workplace wellness, Health, Productivity loss, Depression, Mental illness, Headaches

## Abstract

**Background:**

Historically, in an effort to evaluate and manage the rising cost of healthcare employers assess the direct cost burden via medical health claims and measures that yield clear data. Health related indirect costs are harder to measure and are often left out of the comprehensive overview of health expenses to an employer. Presenteeism, which is commonly referred to as an employee at work who has impaired productivity due to health considerations, has been identified as an indirect but relevant factor influencing productivity and human capitol. The current study evaluated presenteeism among employees of a large United States health care system that operates in six locations over a four-year period and estimated loss productivity due to poor health and its potential economic burden.

**Methods:**

The Health-Related Productivity Loss Instrument (HPLI) was included as part of an online Health Risk Appraisal (HRA) administered to employees of a large United States health care system across six locations. A total of 58 299 HRAs from 22 893 employees were completed and analyzed; 7959 employees completed the HRA each year for 4 years. The prevalence of 22 specific health conditions and their effects on productivity areas (quantity of work, quality of work, work not done, and concentration) were measured. The estimated daily productivity loss per person, annual cost per person, and annual company costs were calculated for each condition by fitting marginal models using generalized estimating equations. Intra-participant agreement in reported productivity loss across time was evaluated using *κ* statistics for each condition.

**Results:**

The health conditions rated highest in prevalence were allergies and hypertension (high blood pressure). The conditions with the highest estimated daily productivity loss and annual cost per person were chronic back pain, mental illness, general anxiety, migraines or severe headaches, neck pain, and depression. Allergies and migraines or severe headaches had the highest estimated annual company cost. Most health conditions had at least fair intra-participant agreement (*κ* ≥ 0.40) on reported daily productivity loss.

**Conclusions:**

Results from the current study suggested a variety of health conditions contributed to daily productivity loss and resulted in additional annual estimated costs for the health care system. To improve the productivity and well-being of their workforce, employers should consider presenteeism data when planning comprehensive wellness initiatives to curb productivity loss and increase employee health and well-being during working hours.

## Background

With the rising costs of health care, employers are rethinking best practice strategies to keep employees healthy. Because employee health is a factor in company profitability, poor employee health must be compensated for through workplace health plans. When including employee health as a factor in overall company profitability, direct health care costs are analyzed and reported as medical health claims and absenteeism. Direct costs generally exist as traceable data and yield clear, easily measured expenses when investigated independently [1]. Indirect costs are the hidden costs of health conditions and involve the behaviors that influence the overall effectiveness of the organization but are not measured in money paid to medical service providers [2]. When employers examine the indirect costs of employee health, the multifaceted concept of presenteeism is commonly considered [3]. Presenteeism is most often correlated with health and is defined as an individual’s loss of work productivity due to health conditions and their symptoms and disease [4]. Some researchers have reported presenteeism with non-health factors related to work environments [1, 5]. Therefore, a more suitable definition of presenteeism may be a person who is physically present at work but performs at a reduced capacity with “decreased productivity and below-normal work quality” for a variety of reasons [1, 6].

Data to determine the impact of illness on employee productivity can be collected using self-report instruments, archival sources, or a combination of both. Although archived data are preferred because they have higher validity, they are seldom used because there is too much missing data. Therefore, self-report instruments have primarily been used to measure health-related productivity loss [7, 8].

Presenteeism self-report instruments are designed to provide insight into how work performance is influenced by a person’s health [5]. There are many self-report instruments that measure health-related productivity loss, but limitations of these instruments present challenges for researchers [8–10]. Health-related productivity loss is susceptible to critique because of concerns with current methods of presenteeism measurement, such as inadequate validation against objective measures, short recall periods being extrapolated to yearly values, and health impairment constructs being self-reported. However, when measuring presenteeism, self-reported data is the usual method used, and it is assumed that participants are answering the items as honestly as possible.

The main goal of researchers in this area is to define and measure presenteeism. As more research becomes available globally, the definition of presenteeism has been broadened, explored, and measured among diverse populations and workplaces. Although reports on lost productivity and costs of presenteeism show mixed results, increasing evidence suggests that presenteeism is a major occupational health problem. Reports on the prevalence and impact of presenteeism are needed to strategically plan care decisions for employees and organizations. Therefore, the purpose of the current study was to evaluate presenteeism among employees of a large United States health care system that operates in six locations over a 4-year period and estimated loss productivity due to poor health and its potential economic burden.

## Methods

The current study was reviewed by the A.T. Still University-Kirksville, Missouri Institutional Review Board and considered to be minimal risk research and exempt from further review. Employees of a large United States health care system across six states who enrolled in a voluntary employee wellness program were included in the study. Each of the six locations managed by this health care system includes a plethora of health services and joint venture facilities including hospitals, assisted living facilities, nursing care facilities, home care, and hospice services. The participants of this study were employees of this health care system including a variety of job types such as maintenance staff, health care practitioners, specialists, pharmaceutical professionals, technologists, clerical staff, therapists, and executives. While this is not an exhaustive list of employee types, it is a depiction of common jobs found within most large health care systems. The data were limited to location and age of the participant.

The Health-Related Productivity Loss Instrument (HPLI) was used to estimate lost productivity from specific health conditions to calculate the economic burden associated with presenteeism. The HPLI includes the following productivity areas: quantity of work, quality of work, work not done, and concentration. Each productivity area is rated on the amount of time affected by the specific health condition from a minimum of “none of the time” to a maximum of “all of the time” (see Appendix).

As part of their wellness program, employees were prompted annually to complete an online Health Risk Appraisal (HRA) that included the measurement of presenteeism using the HPLI. While completing the HRA, participants were asked to check all health conditions that currently applied to them from a list of the following 22 items: any allergies (including seasonal), any cancer, arthritis, asthma, autoimmune disease, cerebrovascular disease/stroke, chronic back pain, depression, digestive disorder, general anxiety, heart disease, hypertension (high blood pressure), mental illness, migraines or severe headaches, neck pain, other metabolic disease (thyroid, kidney/renal, liver), peripheral vascular disease, pulmonary disease, respiratory (chronic bronchitis, emphysema, or sinusitis), seizure disorders or convulsions, type I diabetes, and type II diabetes. These health conditions were chosen by the health care system for their HRA, and thus, only these were available for inclusion in the HPLI. If one or more health conditions affected the participant, they were then prompted to complete the HPLI for each reported condition. For instance, if a participant had migraine headaches and asthma, the participant completed an HPLI for migraine headaches and an HPLI for asthma. The same series of questions were repeated for any other health conditions affecting the participant. The participants spent an estimated 10 min to complete the HRA plus an additional 2–3 min to complete the HPLI associated with each health condition that was selected. Thus, the total time for a participant to complete the HRA was estimated at approximately 10–20 min depending on the number of health conditions selected.

Because recall errors are common in self-reported data and characteristic of instruments that require the reporting of time- or effort-related data [11], participants were asked to consider their work activities before answering the HPLI questions about how each health condition affected their productivity. For instance, they were asked to visualize their work experience for the past 2 weeks, considering accomplishments, work environment, possible work failure, and overall performance level.

In addition to encouraging active memory recall, the length of the recall period was considered to minimize memory errors. Recall periods for self-reported productivity measurements vary among instruments, but it is generally believed that shorter recall periods are more accurate than longer ones [4]. Boles, Pelletier, and Lynch [5] found that 4-week recall periods have considerable recall bias compared with 1-week recall periods. In the current study, a 2-week recall was used with the HPLI, so participants had a suitable period to observe impairments while avoiding recall biases.

To minimize the number of duplicate responses caused by how the health care system of the current study managed new hires, a period was defined as the time from April 1 of 1 year to March 31 of the following year. Data from a 4-year period from April 2011 through March 2015 were included. Period 1 is April 2011 to March 2012, period 2 is April 2012 to March 2013, period 3 is April 2013–March 2014, and period 4 is April 2014 to March 2015. The first HRA a participant completed per period was identified for analysis for that period. A participant completed one HRA each period, with few completing two. Date of birth was also obtained if they completed an HPLI, which we used to calculate age. No other demographic data were available. To obtain an estimate of productivity loss in minutes for each of the four productivity areas included in the HPLI, the midpoint of the time range listed with each response was used (Table 1). Cronbach *α* was used to assess the internal consistency of the HPLI in these four productivity areas for each period and health condition.

**Table 1:** Assignment of productivity loss time categories

Time category	Midpoint
None of the time On average 0 h per day	0
A little of the time On average, less than 1 h per day	30
Some of the time On average, 1–2.9 h per day	119.5
A lot of the time On average, 3–4.9 h per day	239.5
Most of the time On average, 5.0–6.9 h per day	359.5
All of the time On average, 7 or more hours per day	450

The mean productivity loss in minutes across the HPLI’s four productivity areas resulted in an overall estimate for each participant for that specific health condition in each period. Cost calculations assumed an 8-h work day and 240-day work year. Because the data were collected at six individual health care system locations, the hourly wages were based on the local system’s mean hourly rate for that year, i.e., for each year of data, each location had its own hourly rate used in calculations. This was the most granular data available to estimate health productivity and economic burden. Hourly wages and minutes of daily productivity loss for each person were then used to calculate the annual cost per person for each condition. Across the four periods, the mean number and prevalence, estimated as the percent of participants affected by each condition, were calculated. Estimates of daily minutes of productivity loss per person, annual cost per person, and annual company cost were reported with their standard errors by fitting marginal models using the generalized estimating equations approach with an exchangeable working correlation structure to model the correlation of repeated responses from participants while controlling for period. Differences between periods in the population means of minutes of daily productivity loss and annual cost per person for each condition were also tested. Post hoc tests used a Tukey multiple comparison adjustment for the six pairwise comparisons (1 vs 2, 1 vs 3, 1 vs 4, 2 vs 3, 2 vs 4, and 3 vs 4) of periods within a condition. Each health condition was analyzed separately because participants were asked a series of questions to assess presenteeism due to a specific health condition independently of any co-morbidities. For assessing location-specific differences in daily productivity loss, we restricted to conditions with at least 1000 average participants each year to allow for adequate sample sizes.

To assess the agreement within each participant on reported minutes of daily productivity loss from presenteeism across the four periods, *κ* statistics were used. For each condition, responses were categorized as (1) unaffected, zero minutes lost, (2) affected, zero minutes lost, or (3) affected, greater than zero minutes lost. This method distinguished two types of zero productivity loss. An overall *κ* was calculated as a weighted average of *κ* from each category [12]. The *κ* values were interpreted as follows: 0.75–1.00, excellent agreement beyond chance; 0.40–0.75, fair to good agreement beyond chance; and < 0.40, poor agreement beyond chance [12]. The intraclass correlation coefficient is a popular indicator of interrater agreement of continuous data, but because of an inflation of zero responses, *κ* was chosen as a more appropriate measurement for the current study. *P* ≤ .05 were considered statistically significant. Estimates of productivity loss in minutes and costs in dollars were calculated with associated standard errors (SEs) and 95% confidence intervals (CIs). Analyses were conducted using SAS version 9.4 (SAS Institute Inc., Cary, NC).

## Results

After excluding 59 HRAs from 57 employees, their second appraisal within the same period, there were 58 615 HRAs from 23 008 employees collected over the four periods. Data were excluded for 115 (0.5%) employees, who collectively completed 316 HRAs, because their total productivity loss from all indicated health conditions was greater than 8 h per day on at least one HRA. Within the HRA, 26 health condition records were excluded because they indicated 7.5 h of productivity loss per day. Thus, 58 299 HRAs from 22 893 participants were analyzed. Of these participants, 15 933 completed at least one HPLI where the mean age was 45.0 (SD = 12.4, minimum = 16.9 and maximum = 85.8) and 7 959 (34.8%) participants completed an HRA in all four periods. An HRA was completed in three periods by 3 443 (15.0%) participants, in two periods by 4 643 (20.2%) participants, and in one period by 6 848 (29.9%) participants. In each period, the number of participants who completed an HRA ranged from 14 317 to 14 843, and 59.6 to 62.9% of these indicated at least one of the 22 health conditions listed in the HPLI applied to them. Of these participants, the average number of health conditions selected for each period was 1.9 with all periods sharing a minimum of one and a maximum of 11 to 13 conditions, depending on the period.

The internal consistency of the HPLI’s four areas of productivity loss for each of the 22 health conditions is presented in Table 2. The mean Cronbach *α* was highest for seizure disorders (0.91), and most conditions were greater than 0.70, except for arthritis (0.65), cerebrovascular disease/stroke (0.64), heart disease (0.69), and pulmonary disease (0.62). For cerebrovascular disease/stroke and peripheral vascular disease, Cronbach *α* was not estimable for all four periods because there was no variation in minutes for at least one of the productivity areas.

**Table 2:** Internal consistency of minutes of productivity loss for the four productivity areas by medical condition

Medical condition	Cronbach *α*
Any allergies (including seasonal)	0.73 (0.71–0.75)
Any cancer	0.78 (0.69–0.88)
Arthritis	0.65 (0.61–0.68)
Asthma	0.84 (0.81–0.90)
Autoimmune disease	0.88 (0.86–0.91)
Cerebrovascular disease/stroke^a^	0.64 (0.51–0.77)
Chronic back pain	0.77 (0.73–0.81)
Depression	0.85 (0.80–0.89)
Digestive disorder	0.82 (0.75–0.85)
General anxiety	0.84 (0.82–0.85)
Heart disease	0.69 (0.38–0.84)
Hypertension (high blood pressure)	0.77 (0.62–0.85)
Mental illness	0.85 (0.80–0.88)
Migraines or severe headaches	0.87 (0.85–0.89)
Neck pain	0.74 (0.67–0.85)
Other metabolic disease (thyroid, kidney/renal, liver)	0.87 (0.80–0.93)
Peripheral vascular disease^b^	0.78 (0.67–0.99)
Pulmonary disease	0.62 (0.33–0.82)
Respiratory (chronic bronchitis, emphysema, or sinusitis)	0.90 (0.82–0.96)
Seizure disorders	0.91 (0.88–0.94)
Type I diabetes	0.71 (0.20–0.92)
Type II diabetes	0.87 (0.82–0.91)

Data are presented as mean (minimum to maximum) for the four yearly periods

^a^Cronbach *α* was not estimable for all four periods because there was no variation in minutes for at least one of the productivity areas. Only two periods were assessed for this condition

^b^Cronbach *α* was not estimable for all four periods because there was no variation in minutes for at least one of the productivity areas. Only three periods were assessed for this condition

Controlling for the period, for each medical condition, the number and prevalence as percent of affected employees, estimated mean minutes of daily productivity loss per person, annual cost per person, and annual company cost are presented in Table 3. Any allergies was the most prevalent health condition, which affected 4026 (27.6%) participants, followed by hypertension (high blood pressure), which affected 3208 (22.0%) participants. Pulmonary disease was the least prevalent, affecting 21 (0.1%) participants. For minutes of daily productivity loss per person, chronic back pain (16.7 min), mental illness (16.6 min), and general anxiety (16.2 min) ranked highest. Mental illness had the greatest estimated annual cost per person at $2100, and any allergies had the greatest estimated annual company cost at $2.88 million.

**Table 3:** Mean health-related productivity loss instrument outcomes for presenteeism across the four yearly periods

Medical condition	Affected, No. (%)	Estimated daily productivity loss per person, min^a^	Estimated annual cost per person, $^a^	Estimated annual company cost, $^a^
Chronic back pain	631 (4.3)	16.7 (0.84)	1 920 (98)	1 210 000 (62 000)
Mental illness	44 (0.3)	16.6 (3.28)	2 100 (480)	94 000 (21 000)
General anxiety	743 (5.1)	16.2 (0.75)	1 920 (93)	1 430 000 (69 000)
Migraines or severe headaches	1 192 (8.2)	14.7 (0.60)	1 690 (70)	2 020 000 (83 000)
Neck pain	477 (3.3)	11.2 (0.68)	1 290 (80)	610 000 (38 000)
Depression	862 (5.9)	9.9 (0.57)	1 150 (66)	990 000 (57 000)
Autoimmune disease	283 (1.9)	9.5 (1.14)	1 090 (130)	310 000 (37 000)
Arthritis	1 124 (7.7)	8.2 (0.42)	930 (48)	1 040 000 (54 000)
Type I diabetes	78 (0.5)	6.5 (1.54)	710 (170)	56 000 (13 000)
Any allergies (including seasonal)	4 026 (27.6)	6.2 (0.19)	716 (23)	2 880 000 (92 000)
Respiratory (chronic bronchitis, emphysema, or sinusitis)	186 (1.3)	5.4 (0.88)	630 (106)	117 000 (20 000)
Digestive disorder	543 (3.7)	5.3 (0.47)	600 (54)	327 000 (30 000)
Pulmonary disease	21 (0.1)	4.1 (1.57)	450 (170)	10 000 (3 600)
Any cancer	285 (2.0)	3.4 (0.61)	410 (75)	118 000 (21 000)
Asthma	1 017 (7.0)	3.1 (0.26)	350 (30)	350 000 (31 000)
Type II diabetes	887 (6.1)	2.4 (0.29)	270 (33)	244 000 (29 000)
Seizure disorders	59 (0.4)	2.0 (0.87)	240 (103)	14 000 (6 000)
Heart disease	323 (2.2)	2.0 (0.36)	230 (42)	75 000 (14 000)
Other metabolic disease (thyroid, kidney/renal, liver)	948 (6.5)	1.6 (0.20)	179 (23)	169 000 (22 000)
Hypertension (high blood pressure)	3 208 (22.0)	1.1 (0.09)	126 (10)	400 000 (33 000)
Peripheral vascular disease	38 (0.3)	0.9 (0.41)	90 (48)	3 600 (1 800)
Cerebrovascular disease/stroke	34 (0.2)	0.8 (0.36)	90 (38)	2 900 (1 300)

Data are presented in descending order of mean minutes of estimated daily productivity loss per person

^a^Data are reported with associated standard error

**Fig. 1:**
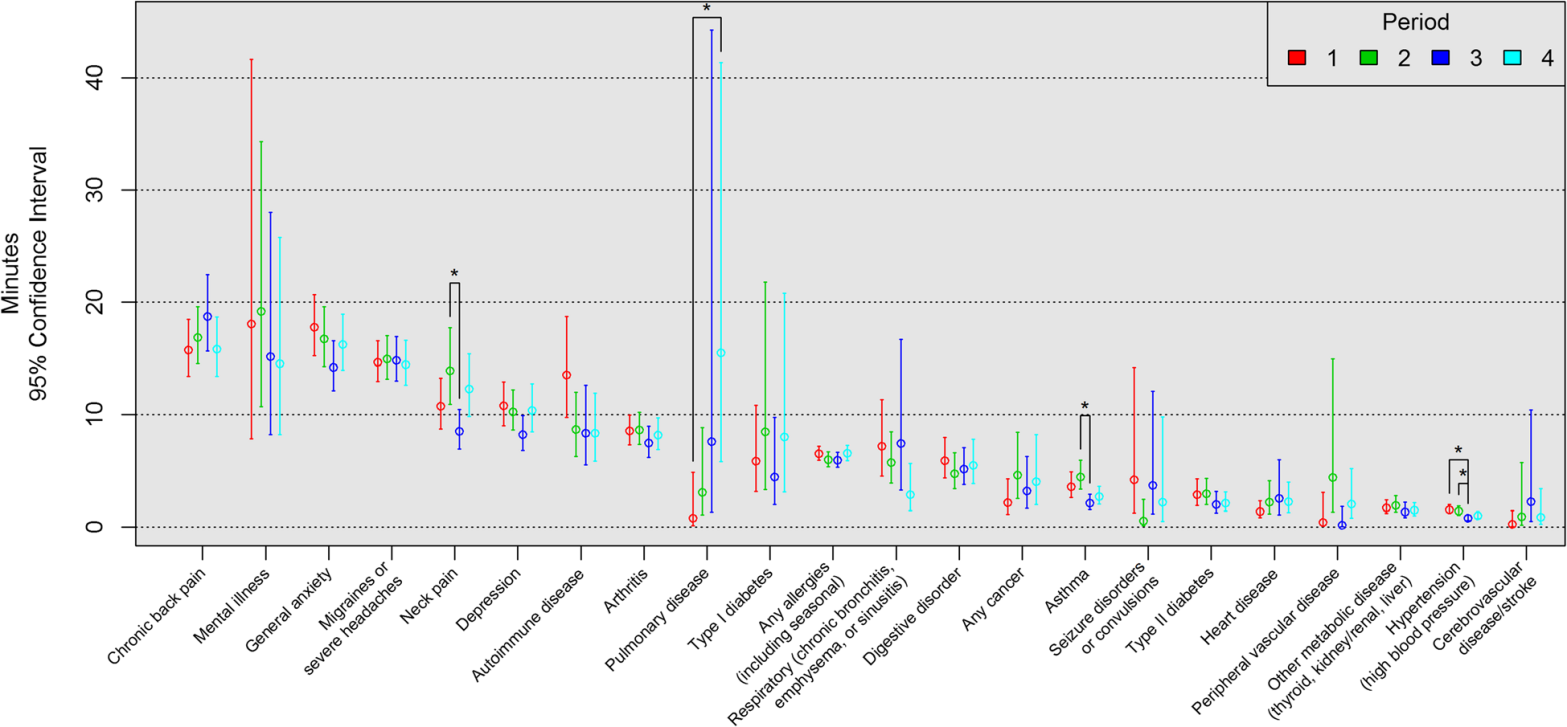
Daily Productivity Loss Per Person (min) from Presenteeism by Period. *Tukey adjustment *P* <.05

**Fig. 2:**
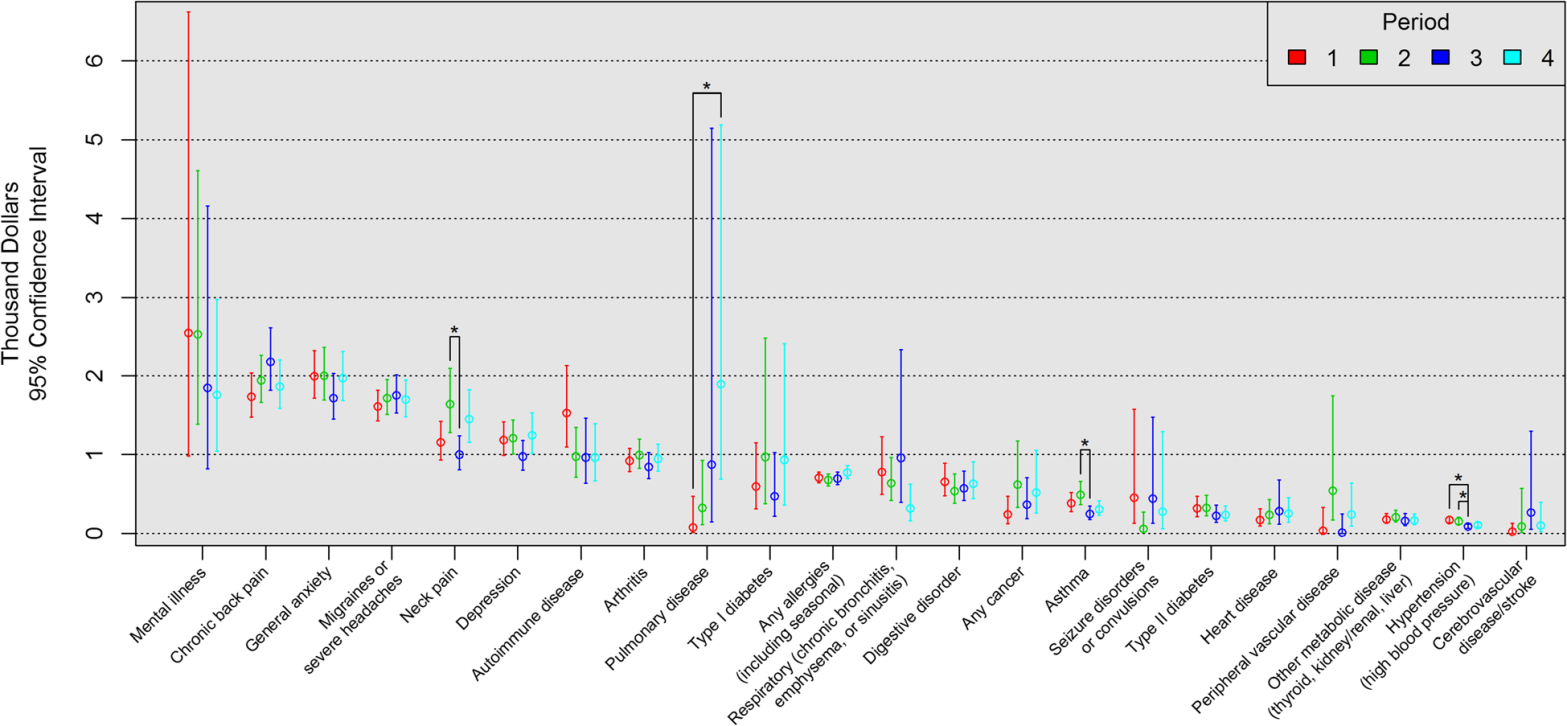
Annual Cost Per Person ($) from Presenteeism by Period. *Tukey adjustment *P* <.05

Daily productivity loss and annual cost per person for each period and condition are shown in Figs. 1 and 2, respectively. In order, chronic back pain, mental illness, and general anxiety ranked highest for daily productivity loss per person by period (Fig. 1), and mental illness, chronic back pain, and general anxiety ranked highest for annual cost per person by period (Fig. 2). For both outcomes by period (Figs. 1 and 2), asthma and neck pain had significantly greater minutes of daily productivity loss (2.3 and 5.4 min, respectively) and annual costs per person ($245 and $638, respectively) in period 2 than in period 3. Hypertension had significantly greater minutes of daily productivity loss and annual costs per person in periods 1 (0.80 min, $85) and 2 (0.60 min, $65) than in period 3. Pulmonary disease had significantly greater minutes of daily productivity loss (14.7 min) and annual costs per person ($182) in period 4 than in period 1.

Among the most prevalent health conditions: any allergies, hypertension, migraines or severe headaches, and arthritis and asthma, all varied in the estimated daily productivity loss per person by location (all *P* < .001).

The overall *κ* and the *κ* for each of the three categories are presented in Table 4. For the overall *κ*, type II diabetes (0.81), hypertension (0.77), asthma (0.74), seizure disorders (0.73), and type I diabetes (0.69) had excellent to good agreement across the periods within each participant on reported minutes of daily productivity loss from presenteeism. These five health conditions also had excellent agreement for the unaffected category (range, 0.85–0.78). By category, agreement was better for the unaffected category than either of the affected categories for all conditions, except for cerebrovascular disease/stroke where the affected, zero minutes lost category was slightly higher (*κ* = 0.48) than the unaffected category (*κ* = 0.47). For the affected, greater than zero minutes lost category, all health conditions had poor agreement, except for type I diabetes (0.50), autoimmune disease (0.40), migraines or severe headaches (0.42), and mental illness (0.43), which had fair agreement.

**Table 4:** Consistency of reported productivity loss from presenteeism across the four periods by medical condition

Medical condition	*κ* statistic				
	No.	Overall	Unaffected, 0 min	Affected, 0 min	Affected, > 0 min
Type II diabetes	22 893	0.81	0.85	0.80	0.36
Hypertension (high blood pressure)	22 892	0.77	0.80	0.78	0.23
Asthma	22 893	0.74	0.80	0.72	0.36
Seizure disorders	22 893	0.73	0.76	0.72	0.38
Type I diabetes	22 893	0.69	0.78	0.64	0.50
Heart disease	22 892	0.61	0.64	0.61	0.29
Other metabolic disease (thyroid, kidney/renal, liver)	22 892	0.60	0.63	0.60	0.26
Autoimmune disease	22 891	0.58	0.66	0.55	0.40
Depression	22 893	0.54	0.62	0.51	0.35
Migraines or severe headaches	22 889	0.54	0.64	0.47	0.42
Mental illness	22 893	0.53	0.58	0.51	0.43
Arthritis	22 892	0.51	0.59	0.47	0.32
Any cancer	22 887	0.49	0.52	0.50	0.08
Cerebrovascular disease/stroke	22 892	0.46	0.47	0.48	0.12
Any allergies (including seasonal)	22 890	0.44	0.51	0.40	0.28
Pulmonary disease	22 893	0.44	0.47	0.46	0.11
General anxiety	22 893	0.42	0.49	0.37	0.33
Chronic back pain	22 891	0.40	0.48	0.33	0.32
Digestive disorder	22 892	0.39	0.43	0.36	0.32
Peripheral vascular disease	22 893	0.34	0.38	0.31	0.20
Neck pain	22 890	0.32	0.39	0.28	0.23
Respiratory (chronic bronchitis, emphysema, or sinusitis)	22 893	0.28	0.31	0.26	0.20

Data are presented in descending order of overall *κ*

## Discussion

Over a 4-year period, 22 health conditions were studied for their effect on daily productivity of employees at a large health care system. The health conditions ranked highest for estimated daily productivity loss per person were chronic back pain (16.7 min), mental illness (16.6 min), and general anxiety (16.2 min). Those most prevalent included any allergies (27.6%), hypertension (high blood pressure) (22.0%), migraines or severe headaches (8.2%) and arthritis (7.7%). The combinations of these factors contributed to five health conditions costing at least $1 million each in estimated annual health care costs from presenteeism and included arthritis ($1.04 million), chronic back pain ($1.21 million), general anxiety ($1.43 million), migraines or severe headaches ($2.02 million), and any allergies ($2.88 million).

The 2014 National Health Interview Survey reported that, among full-time employed adults, 5.9% have asthma [13]. The current study found a similar 7.0% were affected. Other respiratory diseases reported from the National Health Interview Survey included chronic bronchitis at 2.9%, emphysema at 0.6%, and sinusitis at 11.3% [13]. While a person could be represented in more than one respiratory disease category in the National Health Interview Survey, the current study included all employees who had any of these respiratory health conditions in the single respiratory category and found only a collective 1.3% were affected.

When comparing the HPLI to other instruments that measure presenteeism, there are some commonalities and differences in the items chosen for collecting data related to productivity and loss. In 2007, Schultz and Edington [14] conducted a systematic review of presenteeism instruments. The authors [14] found the common attributes of presenteeism measurements included self-reported data regarding one or more health conditions and the effect of such on productivity. Some instruments were designed to single out one health condition, such as migraines, with a focus on productivity time lost but not monetary burden [14]. In a review of presenteeism instruments by Loffland, Pizzi, and Frick [15], 11 instruments were evaluated to specifically determine if valid information could be translated to monetary burden. Of these 11 instruments, only six met the criteria for measuring monetary burden [15]. Given these results, presenteeism instruments appear to be varied, and some are even tailored to one health condition. Further, presenteeism is being measured globally with instruments developed and used in workplaces in a variety of countries, such as Sweden, Denmark, the Netherlands, and Japan [8]. The array of instruments and selected items used to assess presenteeism continue to evolve. Therefore, when assessing presenteeism, many variables related to health and work environments must be considered since work culture affects work productivity. Globally, work culture differs as does work environment; however, health conditions remain a constant in the pursuit of measuring presenteeism.

The rationale for using four productivity areas in the HPLI was to acknowledge an employee’s productivity is not either *on* or *off.* There are degrees of productivity that may be influenced by a health condition. For instance, an individual unable to work for an average of 30 min may report an effect in all four productivity areas, while another individual whose productivity is halved may report an effect only for quality of work and concentration instead of for all four areas. The degree to which participants consistently reported productivity loss across the four areas was measured by Cronbach *α*. Results across the 4-year period for most health conditions assessed were greater than the suggested value of 0.70 given by Nunnally and Berstei [16]. The only health condition to not reach this cut-off value in any period was arthritis, suggesting this health condition may not affect these four productivity areas as consistently as the other health conditions. One possible explanation for the low internal consistency in all four periods for those affected by arthritis is the diversity of employment roles within the health care system, where job requirements vary by the amount of time joints need to be used. For example, work such as typing and operating machinery may see more negative influences on quantity of work than work that involves managing and directing. Further, this collective group of employees who suffer from arthritis may be similar on the quality of work, work not done, and concentration impact productivity areas.

As instruments emerge that include measures of productivity, variables within the workplace and even location may become factors affecting the type and amount of health conditions reported. Seasonal allergies may be more prevalent in a humid climate, and chronic back pain may be exacerbated by physical and laborious tasks or poor ergonomics of working conditions. Nonetheless, all instruments included in systematic reviews [14, 15] suggested that presenteeism is a relevant item to evaluate and that health conditions suffered during work time are associated with lower productivity.

In the current study, asthma, neck pain, hypertension, and pulmonary disease had statistically significant differences between periods for daily productivity loss and annual cost per person. The asthma and neck pain differences were found between period 2 and period 3. Hypertension differences were found from period 1 and period 2 to period 3. Pulmonary disease differences were found between period 4 and period 1. It is unclear whether these observed differences are linked to any cause, but one may be a better managed treatment plan. This is the ideal case. However, no strategic efforts made by the health care system that might explain these reduced effects in any of these conditions are known. Another reason for these differences may be a result of the available employees. The estimates depended on the employees currently employed by the health care system who were willing to participate in the wellness program for that period; therefore, these observed changes may only indicate a change in the population of employees in this wellness program.

Most health conditions in the current study had at least fair intra-participant agreement (*κ* ≥ 0.40) on reported minutes of daily productivity loss from presenteeism, and over half had good to excellent agreement (*κ* > 0.50). High levels of agreement may be attributed to consistency in reporting whether a health condition affected the participant. Type II diabetes, hypertension, asthma, seizure disorders, and type I diabetes had the highest level of agreement (i.e., excellent agreement) for the unaffected category (i.e., when evaluating whether the condition affected the participant), suggesting that these conditions are more chronic. Type I diabetes, mental illness, migraines or severe headaches, and autoimmune disease had the highest level of agreement (i.e., fair agreement) for the affected, greater than zero minutes lost category (i.e., when evaluating whether productivity was negatively affected by the condition), suggesting these conditions most consistently cause productivity loss in employees.

Poor agreement for some health conditions may be a result of the study design. The HRA was completed at various points within the defined yearly period; therefore, an employee with seasonal allergies would have different responses about this health condition depending on when the HRA was completed. Even though any allergies were the most prevalent health condition, agreement for this condition was fair overall and poor when evaluating whether productivity was negatively affected.

In addition to HRA data, the results showing a link between presenteeism and productivity loss have implications for the type and amount of education or health initiatives that can be implemented comprehensively to increase good health choices by employees and also benefit the health care system as a whole. These data may provide new avenues for employers to create more effective health promotion initiatives for work populations and organizations. Employees with chronic or troubling health conditions may benefit from targeted information and action regarding the illness. In addition, education regarding presenteeism and its possible effects on human error is an issue that each individual and organization should be concerned about as it relates to health conditions, safety, productivity, and productivity loss. Further, poor health conditions can have a chronic effect on the ability of employees to safely do their job and can also affect coworkers and subsequent productivity of the company.

The current study had several limitations. Only employees who volunteered to take the HRA were included in the study. As such, volunteers could introduce self-selection bias and since this was part of a wellness program, participants may have been healthier than the general population of employees of large health care systems. This limits the generalizability of the study results to the general population of employees of large health care systems in the United States. Further, participants may not have responded truthfully; however, with any self-reported data, the assumption is that the participants will answer the items honestly. Another limitation is that volunteer overtime work by employees to make up for productivity loss from presenteeism during the 8-h work day was not considered. Further, the HPLI used for the collection of data did not address the overlap of health conditions. For employees with multiple health conditions that increase productivity loss from presenteeism, responses to HPLI items which request separate effects for each condition may be inflated because employees could not separate the effect of other conditions. Additional research is needed to assess the impact of experiencing multiple versus isolated health conditions on the HPLI’s reliability. The current study was designed to determine the annual costs of presenteeism, but it was assumed a 2-week recall period for employees would be a reasonable reflection of the entire year. However, the strength of this assumption may depend on the health condition (e.g., seasonal allergies do not have a consistent effect throughout the year). Finally, for each year of data, each location had its own hourly rate used in calculations. Since wages vary among employees even within the same location and year, the cost attributed to presenteeism for each individual may be over or underestimated depending on the individual’s wage relative to the average. Our results may be overestimated if low-wage earners experience greater daily productivity loss. Future research is needed to assess the distribution of presenteeism across wage levels.

## Conclusions

In the current study, presenteeism was measured using self-reported data from employees of a large health care system over a four-year period. Results suggested a variety of health conditions contributed to daily productivity loss and resulted in additional annual costs for the company. When planning strategic health initiatives, employers should consider both prevalent and expensive health conditions to support health care decisions in the workplace. Globally, presenteeism is measured using various instruments, and the outcomes of these data are used to address indirect costs of productivity. Although many variables and limitations must be considered when reviewing presenteeism data, a convincing argument can be made that presenteeism has great significance when designing targeted health promotion programs and factoring indirect cost into the total economic burden as it relates to employee health. Presenteeism data can be used to support comprehensive efforts to promote wellness in individuals and justify the need for well-designed health promotion initiatives in the workplace.

## Abbreviations

HPLI: Health-Related Productivity Loss Instrument
HRA: Health Risk Assessment
